# Detection of Gram-negative bacterial outer membrane vesicles using DNA aptamers

**DOI:** 10.1038/s41598-019-49755-0

**Published:** 2019-09-11

**Authors:** Hye-Su Shin, Vinayakumar Gedi, Joon-Ki Kim, Dong-ki Lee

**Affiliations:** Department of Chemistry, Sungkyunkwan University, Suwon, 16419 Korea

**Keywords:** Oligonucleotide probes, Bacterial techniques and applications

## Abstract

Infection of various pathogenic bacteria causes severe illness to human beings. Despite the research advances, current identification tools still exhibit limitations in detecting Gram-negative bacteria with high accuracy. In this study, we isolated single-stranded DNA aptamers against multiple Gram-negative bacterial species using Toggle-cell-SELEX (systemic evolution of ligands by exponential enrichment) and constructed an aptamer-based detection tool towards bacterial secretory cargo released from outer membranes of Gram-negative bacteria. Three Gram-negative bacteria, *Escherichia coli* DH5α, *E*. *coli* K12, and *Serratia marcescens*, were sequentially incubated with the pool of random DNA sequences at each SELEX loop. Two aptamers selected, GN6 and GN12, were 4.2-times and 3.6-times higher binding to 10^8^ cells of Gram-negative bacteria than to Gram-positive bacteria tested, respectively. Using GN6 aptamer, we constructed an Enzyme-linked aptamer assay (ELAA) to detect bacterial outer membrane vesicles (OMVs) of Gram-negative bacteria, which contain several outer membrane proteins with potent immunostimulatory effects. The GN6-ELAA showed high sensitivity to detect as low as 25 ng/mL bacterial OMVs. Aptamers developed in this study show a great potential to facilitate medical diagnosis and early detection of bacterial terrorism, based on the ability to detect bacterial OMVs of multiple Gram-negative bacteria.

## Introduction

Bacterial infections can be detrimental due to their virulence factors, underscoring the need for development of rapid, accurate, and sensitive detection techniques^1^. Gram-negative bacteria especially represent an important medical challenge. They are resistant to various antibiotics because their outer membrane evolves and adapts to the protective mechanisms against the selection pressure of antibiotics^2^. Lipopolysaccharides (LPS), a major constituent of the outer membrane of Gram-negative bacteria, induces a strong innate immune response in host environments^3^. Furthermore, outer membrane vesicles (OMVs) with size of 20 to 250 nm, which are commonly produced and secreted from outer membranes of bacteria, carry various bacterial membrane proteins and other virulence factors^4,5^. OMVs are known to trigger severe pathogenesis, enhance bacterial survival, transfer genetic and protein components for cell-free intercellular communication, deliver toxic compounds, and trigger immune response in host cells^6,7^.

Various surface antigens exist on the cell surface of Gram-negative bacteria, which can be potential targets for bacterial detection. These antigenic moieties, including LPS^8,9^, peptidoglycans^10,11^ and lectins^12^ have served as substrates of the bioreceptors. Conventional methods of bacterial detections in general rely upon three types of laboratory-based techniques such as optical assays using fluorescent labeling^13,14^ or surface plasmon resonance^15^, mechanical quartz crystal microbalance sensors^16,17^ and electrochemical sensors^18^ such as potentiometric, amperometric and impedimetric assays. Despite the vast amount of research, sensitive bacterial detection methods which meet commercial demands in real situations are yet to be developed^19^. Detecting living bacteria in blood is difficult due to the immune regulation of host cells and the serum bactericidal activity^20,21^. This suggests that the detection of bacterial OMVs is more effective than the detection of bacterial cells in clinical blood samples^22^.

In this study, nucleic acid aptamers are proposed for the development of inexpensive and rapid diagnostic tools, because aptamers could recognize target epitopes with high selectivity and specificity using the screening technique of systemic evolution of ligands by exponential enrichment (SELEX)^23^. Using cell-SELEX, aptamers can be isolated against target epitopes in its natural physiological context, even when the exact target on the cell surface is unknown^24,25^. Using bacterial cell-SELEX, several aptamers have been isolated against different bacterial species with high affinity and specificity. The dissociation constants (*K*_*d*_) of several reported aptamers are in the nanomolar range with low limit of detection of about 1 to 1000 CFU/mL^26^.

Although most bacterial cell-SELEX studies focus on targeting single bacterial strain, it is possible that aptamers could bind epitopes commonly shared among different bacterial strains^27,28^. A previous study which performed SELEX against a single *E*. *coli* strain demonstrated that the selected aptamers can also bind other strains of *E*. *coli*, implicating that some bacterial strains share common targets on their cell surface^29^. It also means that toggling different targets of interest beyond the conventional SELEX procedure can isolate cross-reactive aptamers with broad range of binding^30,31^. One recent study reported isolation of aptamers against six different genera of bacteria using Toggle-cell SELEX^32^.

Here, we used Toggle-cell SELEX to generate single stranded DNA aptamers which exhibit broad cross-reactivity to different Gram-negative bacterial strains. Three bacteria in Enterobacteriaceae, *Escherichia coli* DH5α, *Escherichia coli* K12, and *Serratia marcescens* were selected in the screening. After four rounds of toggle loops, the selected aptamers were characterized. Two selected aptamers, GN6 and GN12, showed broad binding efficiency to multiple strains of Gram-negative bacteria, but not to Gram-positive bacteria. Using GN6 aptamer, we constructed an Enzyme-linked aptamer assay (ELAA)^33,34^ to detect outer membrane vesicles (OMVs) of Gram-negative bacteria, which contain several outer membrane proteins with potent immunostimulatory effects. This GN6-ELAA platform showed high sensitivity to detect as low as 25 ng/mL bacterial OMVs.

## Results and Discussion

### Gram-negative bacterial Toggle-cell SELEX

**Figure 1:**
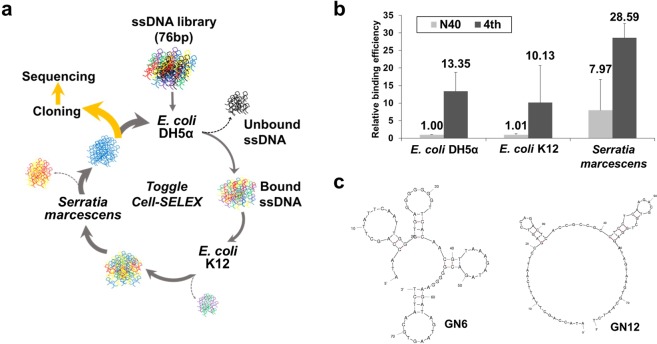
Toggle-Cell SELEX against multiple Gram-negative bacteria. (**a**) Schematic illustration of Toggle-Cell SELEX against three different Gram-negative bacteria. (**b**) Relative binding efficiency was tested using qPCR to confirm the enrichment of ssDNA after 4th round of Toggle SELEX. Data represented mean ± SD values of three independent experiments. (**c**) Predicted secondary structures of two isolated aptamers, GN6 and GN12, using M-fold algorithm at 25 °C, [Na^+^] = 100 mM, and [Mg^2+^] = 5 mM.

Bacterial Toggle-cell SELEX was performed to isolate ssDNA aptamers against multiple Gram-negative bacteria. Three Gram-negative bacteria, *E*. *coli* DH5α, *E*. *coli* K12, and *S*. *marcescens*, were selected for the isolation of aptamers (Fig. 1a). In the first round of selection, *E*. *coli* DH5α cells were incubated with the random 76-nt ssDNA library. Unbound ssDNA mixture was discarded, and only bound ssDNA pool was recovered and amplified by PCR for next enrichment. Next, *E*. *coli* K12 and *S*. *marcescens* were used sequentially in the same procedures. This toggle loop was repeated 4 times until the relative amount of binding, measured using quantitative real-time PCR, of DNA aptamers mixtures was 13.35-, 10.03- and 3.59- times higher than that of the starting DNA library to three bacteria, respectively (Fig. 1b). The final products were amplified and cloned for sequencing analysis. After selection of two DNA sequences, GN6 and GN12, as aptamer candidates (Table 1), their secondary structures were predicted at 25 °C in buffer solutions which contain 5 mM MgCl_2_, 100 mM NaCl using M-fold algorithm (Fig. 1c).

**Table 1:** ssDNA sequences of two isolated aptamers after Gram-negative bacterial Toggle-cell SELEX.

ID	DNA sequence (5′ to 3′)
GN6	ATA CCA GCT TAT TCA ATT GGG TGA GGG GGG GTT CAC AAC GTT AAA GAT AGA CGG GGG AAG ATA GTA AGT GCA ATC T
GN12	ATA CCA GCT TAT TCA ATT CCG AGT CCA GAC TCA CCG CCG CCT CCT CAA GAC GTG CTG GAG ATA GTA AGT GCA ATC T

### Binding affinity of isolated aptamers

**Figure 2:**
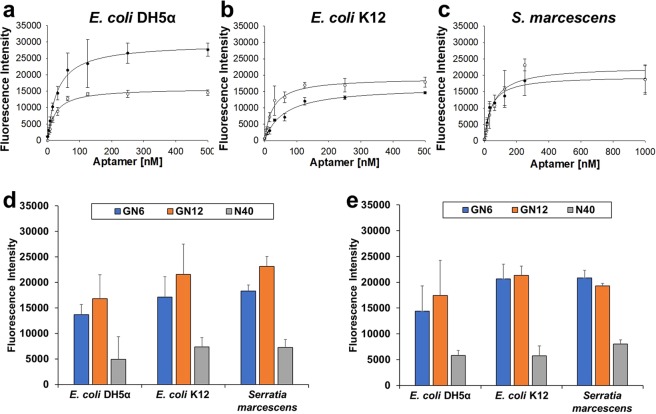
Binding affinity of GN6 and GN12 to three Gram-negative bacteria. Binding saturation curves of both GN6 (●) and GN12 (○) were fitted using non-linear regresson model of SigmaPlot 12.0 towards (**a**) *E*. *coli* DH5α, (**b**) *E*. *coli* K12 and (**c**) *S*. *marcescens*. (**d**) 10^8^ cells and (**e**) 10^5^ cells of bacteria were incubated with the two selected aptamers at 250 nM concentration and N40 library as control. Data represents mean ± SD values of three independent experiments.

Next, we quantified binding affinities of GN6 and GN12 aptamers against three Gram-negative bacteria used in SELEX. Various concentrations of aptamers labelled with FAM (carboxyfluorescein) at the 3′-end were added to 10^8^ cells of bacteria. After measuring the fluorescence signal, the binding saturation curves were fitted using non-linear regression model, based on the following equation: S = B_*max*_ × C/(*K*_*d*_ + C) (where S represents the fluorescence (FAM) intensity, B_*max*_, the maximum binding intensity, *K*_*d*_, the dissociation constant, and C, concentrations of aptamer) (Fig. 2a–c). The dissociation constants (*K*_*d*_) were observed in the range of 20.36 to 59.70 nM against three bacterial strains tested (Table 2). These values were in similar range with previous experimental results in other studies^26^. The binding affinities of two aptamers were compared with that of random ssDNA (N40) at 250 nM concentration in 10^8^ and 10^5^ cells of Gram-negative bacteria, respectively (Fig. 2d,e). Both GN6 and GN12 aptamers showed average 2.55- and 3.19-times higher binding towards 10^8^ cells of bacteria than N40 random sequence. Likewise, it showed average 2.89- and 3.03-times higher binding to 10^5^ cells of bacteria than the same control.

**Table 2:** Dissociation constants (*K*_*d*_) of GN6 and GN12 aptamers against three bacteria.

Aptamer	*E*. *coli* DH5α	*E*. *coli* K12	*S*. *marcescens*
GN6	29.94 ± 2.49 nM	59.70 ± 10.89 nM	38.98 ± 6.46 nM
GN12	20.36 ± 2.38 nM	24.80 ± 3.98 nM	53.83 ± 17.70 nM

### Aptamer cross-reactivity towards multiple Gram-negative bacteria

**Figure 3:**
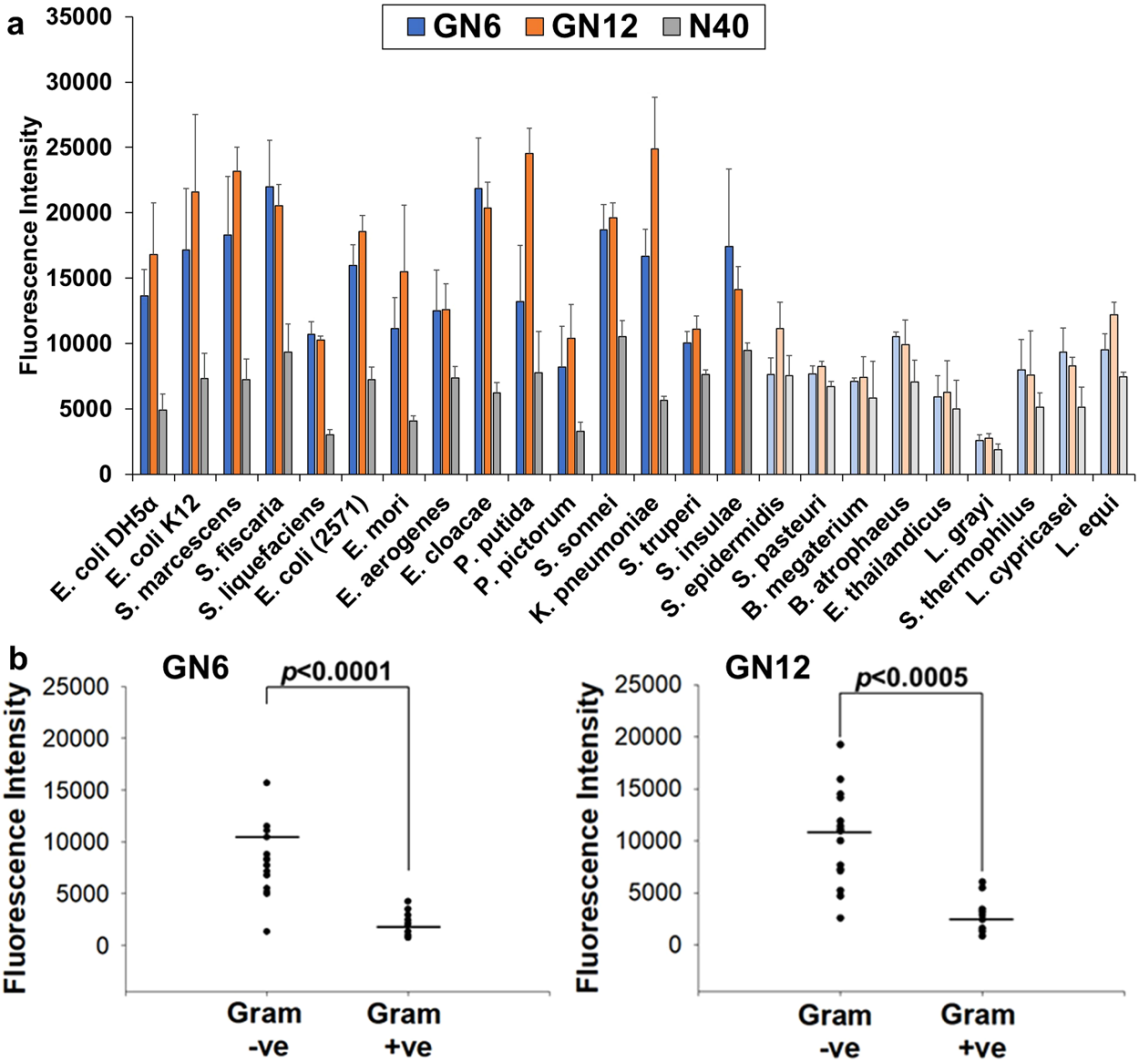
Binding profile of GN6 and GN12 against Gram-negative and positive bacteria. (**a**) Binding assay was performed using GN6 (blue) and GN12 (orange) at 250 nM against 10^8^ cells of multiple Gram-negative (vivid) and Gram-positive (pale) bacteria. Binding efficiencies were estimated by measuring the fluorescence intensity of bound aptamers to bacteria. (**b**) GN6 (left) and GN12 (right) showed 4.2-times and 3.6-times higher binding to 10^8^ cells of Gram-negative bacteria than to Gram-positive bacteria tested, respectively. Data represented mean ± SD values of three independent experiments and *p*-values were analyzed using student’s *t*-test.

Next, the broad cross-reactivity of selected aptamers was analysed by performing binding assay against multiple Gram-negative bacteria, based on the assumption that they share similar structural components as common cell surface epitopes. Both GN6 and GN12 (250 nM) showed high binding to 10^8^ cells of various Gram-negative bacteria tested, including the well-known pathogenic species, *K*. *pneumoniae*, *E*. *cloacae* and *S*. *sonnei* (Fig. 3a). It should be noted that aptamers showed lower binding to one Gram-negative bacteria tested, *S*. *trueperi*, which is a *Sphingomonas* species that belongs to α-proteobacteria^35^. In contrast, significantly reduced binding efficiency was observed against all Gram-positive bacterial strains tested. GN6 aptamer could detect Gram-negative bacteria 4.2-times higher than Gram-positive bacteria (*p* < 0.0001). GN12 aptamer also showed 3.6-times higher binding to Gram-negative bacteria than Gram-positive bacteria (*p* < 0.0005) (Fig. 3b). These broad cross-reactivity and specificity to Gram-negative bacteria were also observed when 10^5^ cells of bacteria were incubated (Fig. S1a). Both GN6 and GN12 aptamer at 250 nM concentration were able to detect Gram-negative bacteria 3.9-times and 3.4-times higher than Gram-positive bacteria, respectively (*p* < 0.0001) (Fig. S1b). Without negative selections against Gram-positive bacteria during Toggle-cell SELEX, selected aptamers showed no binding preference to them, suggesting that the unknown targets of aptamers on cell surface exclusively expressed in Gram-negative bacteria. Future studies are required to identify the specific targets of GN6 aptamers on OMVs surface. Furthermore, we also noticed inconsistency between dissociation constants and binding profiles of both aptamers regarding their binding abilities to Gram-negative bacteria. Higher binding affinity of aptamers with low *K*_*d*_ values generally represents the higher intermolecular interaction between a single target on bacteria and its bound aptamer. In contrast, Fig. 3 showed the maximum binding capacity (B_*max*_) when all targets are fully saturated by excessive amounts of aptamers (250 nM). These two binding parameters, affinity and capacity, can be separately interpreted because capacity is affected by several factors such as multivalent interactions when an aptamer shares multiple antigens of bacteria or the density of targets on bacterial outer membrane^29,36^.

### Isolation and characterizations of bacterial OMVs

**Figure 4:**
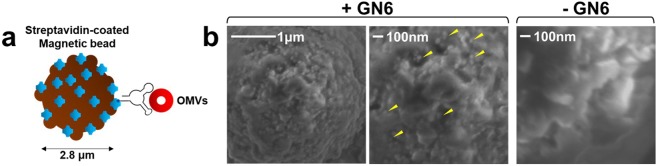
*E*. *coli* DH5α-derived OMVs bound to GN6 aptamer. (**a**) Scheme of Bead-GN6-*E*. *coli* DH5α OMVs complex. (**b**) SEM images of *E*. *coli* DH5α-derived OMVs (yellow arrow) bound to GN6 aptamer. Without GN6 aptamer, no OMVs were shown.

It has been known that OMVs budding out from outer membranes of Gram-negative bacteria carry several virulence biomolecules and endotoxins^7^. We isolated bacterial OMVs using ultracentrifugation. DLS analysis exhibited the size distribution of OMVs ranging from 84.29 to 176.4 nm (Table 3), within the range of general agreement^4,5^. There are no general bacterial OMV markers, but OMV proteins such as OmpA (35.2 kDa) in *E*. *coli*^37^ and Serralysin (~52–55 kDa) in *S*. *marcescens*^38^ could be used for characterization (Fig. S2). Next, magnetic bead-pull down assay using streptavidin-coated beads and 3′-biotinylated GN6 aptamer was performed to capture OMVs^39^ (Fig. 4a). The binding between *E*. *coli* DH5α OMVs and GN6 was visualized by scanning electron microscope (SEM) (Fig. 4b). Without GN6 aptamer, spherical nanoparticles of *E*. *coli* DH5α OMVs were not shown. It suggests that the targets of GN6 on *E*. *coli* DH5α are also present in *E*. *coli* DH5α OMVs. This leads us to develop an aptamer-based detection platform for bacterial OMVs.

**Table 3:** Size evaluations of bacterial OMVs via DLS.

	Z-average (diameter, nm)	PDI (Polydispersity index)
*E*. *coli* DH5α	105.9	0.455
*E*. *coli* K12	97.96	0.420
*S*. *marcescens*	164.4	0.335
*L*. *grayi*	176.4	0.490
*B*. *megaterium*	84.29	0.410

### ELAA platform for detecting Gram-negative bacterial OMVs

**Figure 5:**
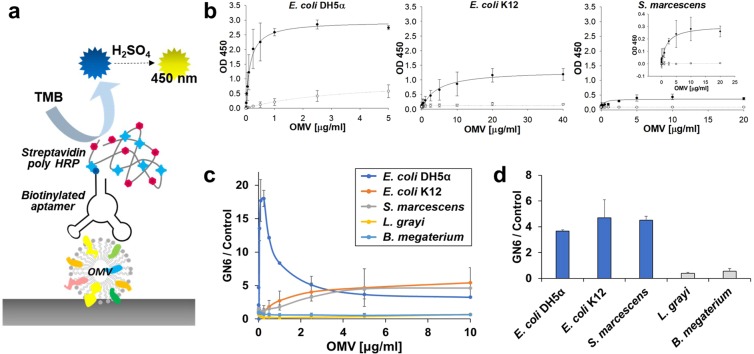
GN6-ELAA for Gram-negative bacterial OMVs. (**a**) Scheme of GN6 ELAA for bacterial OMVs. (**b**) Three Gram-negative bacterial OMVs showed highly sensitive binding to GN6 aptamer (●) rather than N40 control (○) at 250 nM concentration. (**c**) GN6 ELAA towards both Gram-negative and Gram-positive bacterial OMVs. (**d**) GN6 ELAA showed higher specificity to 5 μg/mL of Gram-negative bacterial OMVs compared to 5 μg/mL of Gram-positive bacterial OMVs from *L*. *grayi* and *B*. *megaterium*. Data represented mean ± SD values of three independent experiments.

Using the selected aptamer, GN6, we have developed an Enzyme-linked aptamer assay (ELAA) to detect OMVs originated from various Gram-negative bacteria. Instead of conventional ELISA using the detection antibody conjugated with HRP (Horse radish peroxidase), GN6 aptamer was used as a bait (Fig. 5a). GN6 ELAA showed highly recognizable signals against three Gram-negative bacterial OMVs. The dissociation constants of GN6 to OMVs derived from *E*. *coli* DH5α, *E*. *coli* K12 and *S*. *marcescens* were 0.13 ± 0.01 μg/ml, 3.70 ± 0.98 μg/ml and 0.23 ± 0.16 μg/ml, respectively (R^2^ = 0.99) (Fig. 5b). It especially showed the highest binding affinity and capacity to *E*. *coli* DH5α OMVs. Meanwhile, this assay also showed high sensitivity, which could detect as low as 25 ng/mL of Gram-negative bacterial OMVs. Consistent with GN6 binding affinity to bacterial cells, it showed the similar pattern to binding affinity to bacterial OMVs, strongly suggesting that target of GN6 aptamers is present both on Gram-negative bacterial cells and on the surface of Gram-negative bacterial OMVs. Consistent with the previous results, OMVs from Gram-positive bacteria, *L*. *grayi* and *B*. *megaterium*, showed no recognizable binding to GN6 (Fig. 5c,d). These results indicate that GN6 ELAA could detect multiple Gram-negative bacteria derived OMVs. It opens the new possibility of developing cell-free bacterial sensor using bacterial OMVs as substrates instead of living bacterial cells.

## Conclusion

While various systems are being developed to detect pathogenic bacteria, few studies have reported the isolation of broadly cross-reactive aptamers against various species of bacteria. Here, we developed highly specific DNA aptamers, GN6 and GN12, against many Gram-negative bacteria, including pathogenic strains. Selected aptamers after Toggle-cell SELEX showed broad cross-reactivity towards many Gram-negative bacteria tested. Using GN6 aptamer, we developed an GN6-ELAA to detect Gram-negative bacterial OMVs from cell-free supernatant. This is because unknown targets of GN6 on the original bacterial outer membrane could also be expressed in the surface of bacterial OMVs. Moreover, the GN6-ELAA had high sensitivity to low concentration of Gram-negative bacterial OMVs and high specificity exclusively bound to them. Further studies will require identification of GN6 aptamer targets and increasing the yield and purity of OMVs. If we increase the final yield and purity of OMVs, it will be possible that the detection of bacterial OMVs in cell-free medium leads to the accurate identification of the originated bacteria. We believe that the aptamer-based Gram-negative bacterial OMV detection has a great potential to facilitate medical diagnosis and early detection of bacterial terrorism.

## Methods

### Bacterial strains and culture

All bacteria were purchased from the Korean Collection for Type Culture (KCTC, Korea). *E*. *coli* DH5α, *E*. *coli* K12, *E*. *coli* (KCTC 2571), *S*. *sonnei*, *K*. *pneumoniae*, *S*. *epidermidis* and *S*. *pasteuri* were cultivated at 37 °C in LB medium, *S*. *marcescens*, *S*. *ficaria*, *S*. *liquefaciens*, *E*. *mori*, *E*. *aerogenes*, *E*. *cloacae*, *B*. *megaterium*, and *B*. *atrophaeus* were grown at 30 °C in NB medium, *P*. *putida*, *P*. *pictorum*, and *S*. *insulae* were grown at 25 °C in LB medium, *E*. *thailandicus*, *S*. *thermophilus*, *L*. *cypricasei*, and *L*. *equi* were grown at 37 °C in Lactobacillus MRS broth, and *L*. *grayi* at 37 °C in BHI medium. All these bacteria were cultured under aerobic conditions up to OD_600_ of 0.4, followed by centrifugation (10,000 rpm) for 10 min at 4 °C, and washing twice with Tris-HCl buffer (50 mM Tris, pH 7.4, 1 mM MgCl_2_, 5 mM KCl, 100 mM NaCl). The washed bacteria were resuspended in binding buffer (50 mM Tris, pH 7.4, 5 mM MgCl_2_, 5 mM KCl, 100 mM NaCl, 1 mg/mL BSA, 0.1 mg/mL Salmon sperm DNA, 0.1 mg/mL yeast tRNA).

### Gram negative bacterial Toggle-Cell SELEX

All oligonucleotides were purchased from Integrated DNA technologies (Coralville, USA). The PAGE-purified single-stranded DNA library consisted of an N_40_ randomized region flanked by two 18-nt primer-binding regions for PCR (5′-ATA CCA GCT TAT TCA ATT-N_40_-AGA TAG TAA GTG CAA TCT-3′). The following forward primer (5′-ATA CCA GCT TAT TCA ATT-3) and reverse primer (5′-AGA TTG CAC TTA CTA TCT-3′) were used for quantitative real-time PCR. The reverse primer modified with poly-A tail overhang (5′-AAA AAA AAAAAAAAAAAA AA/iSp9//iSp9/AGA TTG CAC TTA CTA TCT-3′) was used for PCR in SELEX process. In the first round of SELEX, the ssDNA library pool (2500 pmol) was denatured in binding buffer for 5 min at 95 °C and cooled on ice for 5 min. It was mixed with 10^8^ number of *E*. *coli* DH5α cells in binding buffer with constant shaking at RT for 15 min. The ssDNA bound to *E*. *coli* DH5α was collected at 6,000 rpm for 5 min at 4 °C. The bound ssDNA was separated from bacterial cells by elution in Tris-EDTA buffer (50 mM Tris, pH 8.0, 10 mM EDTA, 1 mM MgCl_2_, 5 mM KCl, 100 mM NaCl) at 95 °C for 5 min and cooled on ice for 5 min. The ssDNA after elution was collected at 13,000 rpm, 4 °C for 5 min, purified using PCI solution (Sigma Aldrich, USA), and precipitated in ethanol including 5 v/v% ammonium acetate and 1.5 v/v% glycogen. The PCR mixtures were made by ssDNA template (~20 ng), 0.4 μM of forward and poly-A tailed reverse primer, MyTaq Reaction buffer, and 0.25 U My Taq DNA polymerase (Bioline, UK). After PCR, the reaction products were separated by 10% PAGE gel in 1X TBE (Tris-borate-EDTA). To separate the interested ssDNA, gel purifications in UREA-PAGE gel were performed using asymmetric poly-A tailed reverse primer. The dsDNA PCR product separated by forward primer in UREA gel was separated, heated at 65 °C for 30 min in elution buffer (10 mM Tris, pH 7.4, 1 mM EDTA, 750 mM NH_4_OAc, 0.1% SDS) and collected in 0.3 M sodium acetate and 0.25 μg/μL glycogen (pH 5.2). Subsequently, 2.5 volumes of 100% ethanol added and incubated at −80 °C for 12 h, followed by centrifugation at 13,000 rpm, 4 °C for 30 min. The DNA pellets were dissolved in water and quantified using BioSpecNano Spectrophotometer (SHIMADZU, Japan). For the next round of selection, 100 pmol of ssDNA from the first round was mixed with 10^8^ cells of *E*. *coli* K12. The following procedure was the same as above. Subsequently, ssDNA was isolated against *S*. *marcescens* and the same procedures were repeated four times (total 12 rounds). After the final round, the isolated ssDNA pools were amplified by RBC T&A cloning vector kit (Real Biotech, Taiwan). ssDNA aptamer candidates were transformed into competent *E*. *coli* DH5α and then plasmid DNA were purified using QIAprep Spin Miniprep Kit (Qiagen Inc., Germany). The secondary structures were computationally predicted using M-fold algorithm (http://mfold.rit.albany.edu) at RT, [Na^+^] = 100 mM, and [Mg^2+^] = 5 mM.

### Binding enrichment test using quantitative real-time PCR

The standard controls were made by serial dilution of samples. Each ssDNA sample was mixed with 0.2 μM of forward, reverse 18-nt primers and SYBR® Premix Ex Taq^TM^ (TliRNaseH Plus, Takara Bio Inc, Japan), followed by relative quantification analysis using StepOne^TM^ Real-Time PCR System (Applied Biosystems®, USA) according to the manufacturer’s protocol.

### Fluorescence-based binding assays for aptamers

The binding affinity and capacity to bacteria were quantified by binding 3′-FAM labelled aptamers to the bacterial cells. For measuring the dissociation constants, 10^8^ cells of bacterial species were bound to different aptamer concentrations at RT for 15 min. To determine whether the aptamers showed non-specific binding to bacteria, the same concentrations of the negative controls as 3′-FAM labeled N40 library were incubated in the same bacteria used above. After incubation, the samples were washed three times in Tris buffer to remove unbound ssDNA, at 10,000 rpm, 4 °C for 10 min. Samples were resuspended in water, and their fluorescence intensity was measured using VICTOR X2 Multilabel Plate Reader (PerkinElmer, USA). The dissociation constant was measured by a non-linear regression fit model of SigmaPlot12.0. The binding efficiency or capacity of the two selected aptamers at 250 nM concentration was measured by incubating 10^8^ and 10^5^ cells of bacteria. It was also compared with that of 3′-FAM-labeled N40 random ssDNA upon incubation with 10^8^ and 10^5^ cells of bacteria.

### Isolation and characterizations of OMVs

Bacterial cultures grown overnight in media were pelleted at 10,000 rpm for 30 min. The supernatant fraction was filtered through a 0.45 μm syringe (Merck Millipore, USA) to remove any remaining cell debris, and concentrated 50-fold by ultrafiltration using 100 kDa Amicon® Ultra-0.5 device (Merck Millipore). One more filtration was performed using 0.22 μm syringe filter (Merck Millipore). Next, OMVs were isolated by ultracentrifugation (Optima MAX-XP, Beckman Coulter, Inc., USA) at 150,000 rpm for 3 h at 4 °C, resuspended in PBS and stored at −80 °C. The protein concentrations were measured using micro BCA assay (Thermo Scientific, USA). To estimate the size distributions of the isolated OMVs, dynamic light scattering (DLS) was carried out using Zetasizer Nano ZS90 (Malvern, UK). Samples were diluted 1:1000 in PBS and processed at 25 °C under standard settings (Dispersant Refractive Index = 1.331, viscosity (cP) = 0.89). To visualize the binding between GN6 and *E*. *coli* DH5α OMVs, 200 μg of streptavidin-coated magnetic beads (Dynabeads™ M-280 Streptavidin, Thermo Scientific, USA) and 50 pmol of GN6 aptamer were mixed for 30 min in mild shaking. After washing once, 10 μg/mL of OMVs were added and incubated for 15 min, followed by washing several times to remove unbound OMVs. These samples were fixed in a 2% paraformaldehyde solution for 2 h and diluted in distilled water, followed by immobilization on the clean silicon chips under drying conditions. To make surface conductive, Au-Pd alloy was applied by sputtering before imaging. SEM using JSM-7100F was performed in 2 or 5 kV of beam energy.

### Aptamer-based direct OMVs detection

For GN6 ELAA using bacterial OMVs, Nunc-Immuno 96 MicroWell solid plates (Thermo Scientific, USA) were used to immobilize bacterial OMVs in Tris buffer. After incubating OMVs at various concentrations in the plate overnight at 4 °C, the plate was washed twice and blocked using 2% BSA-Tris buffer for 2 h. After blocking, 20 pmol of GN6 aptamer and N40 control were separately added and incubated for 1 h. After washing 4 times, streptavidin-Poly HRP conjugate (Pierce, USA) was added and incubated for 30 min. After thoroughly washing 5 times in Tris buffer with 0.05% Tween-20, Ultra TMB-ELISA reagent (Thermo Scientific, USA) was added. After 15 min, 1 M sulfuric acid as stop solution was added. Absorbance at 450 nm was measured using Multiskan microplate photometer (Thermo Scientific, USA). The measured values were analyzed using non-linear regression fit model of SigmaPlot 12.0.

## Supplementary information

Supplementary Data
