# Supplementary material for: Detection of Gram-negative bacterial outer membrane vesicles using DNA aptamers
Source: Sci Rep. 2019 Sep 11;9:13167. doi: 10.1038/s41598-019-49755-0 (PMC6739373; doi:10.1038/s41598-019-49755-0)
Supplement: Supplementary file 1 — Supplementary Data [file 41598_2019_49755_MOESM1_ESM.docx]

**Supplementary Data**

Detection of Gram-negative bacterial outer membrane vesicles using DNA aptamers

Hye-Su Shin, Vinayakumar Gedi, Joon-Ki Kim & Dong-ki Lee

Department of Chemistry, Sungkyunkwan University, Suwon, 16419, Korea

* Corresponding Author. E-mail address: dklee0318@gmail.com


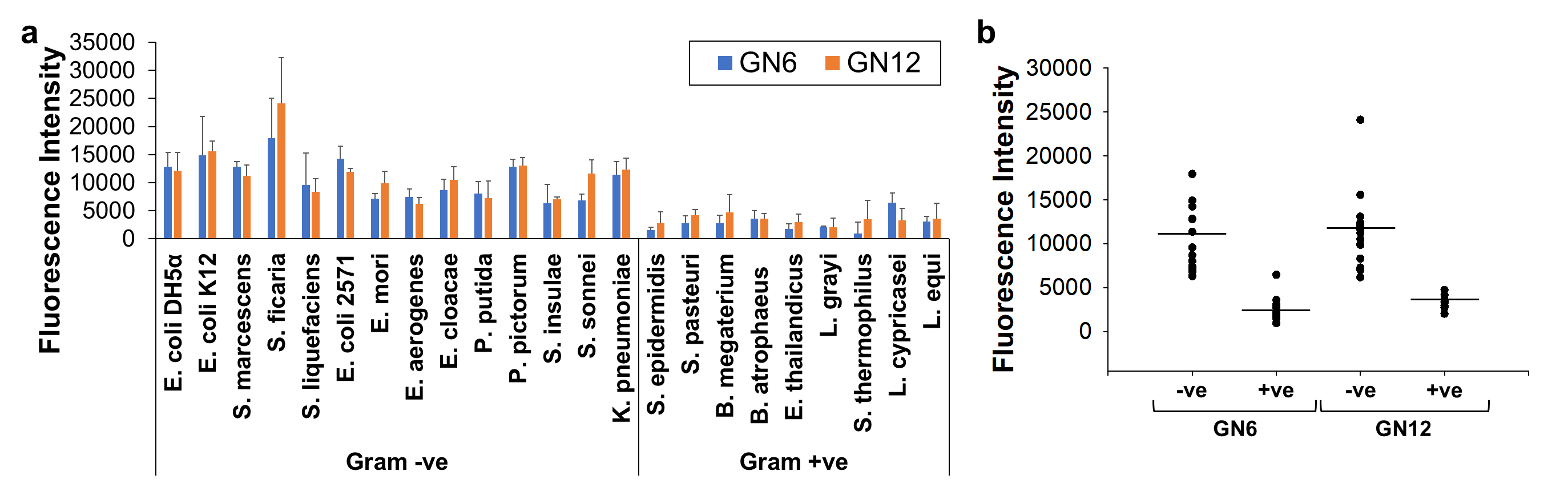


Figure S1. Binding profiles of GN6 and GN12 (250 nM) against 10^5^ cells of Gram-negative and positive bacteria. (A) The binding affinities were estimated by measuring the fluorescence intensity of bound aptamers. Broad cross-reactivity and specificity only to various Gram-negative bacteria were observed. (B) Both GN6 and GN12 aptamers were able to detect Gram-negative bacteria 3.9 times and 3.4 times higher than Gram-positive bacteria, respectively (*p*<0.0001). Data represented mean ± SD values of three independent experiments and *p*-value were analyzed using student’s t-test.


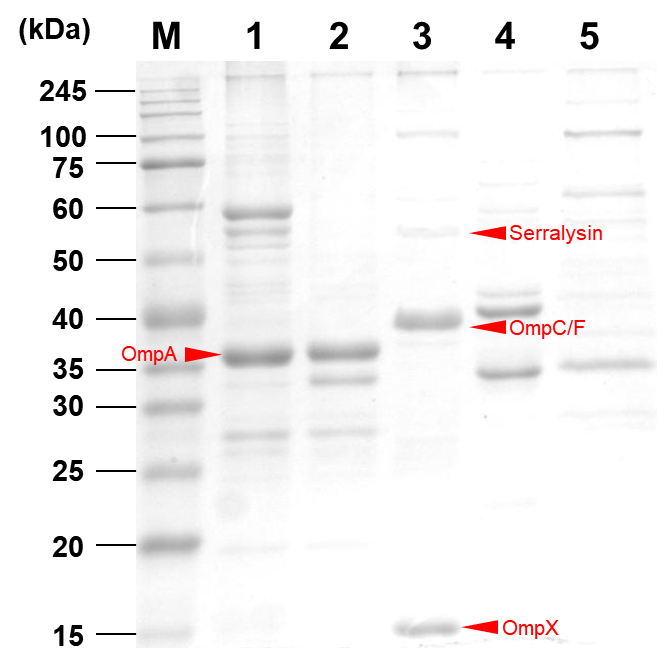


Figure S2. 13% SDS-PAGE separation of five bacterial OMVs (10 μg) with Coomassie blue staining. (M) Protein marker; (lane 1) *E. coli* DH5α; (lane 2) *E. coli* K12; (lane 3) *S. marcescens*; (lane 4) *B. megaterium*; and (lane 5) *L. grayi*.

Outer membrane proteins of Gram-negative bacteria are largely composed of the homologous β-barrel family^1^. Abundant β-barrel OmpA protein (35.2 kDa), especially, is one of the major proteins from outer membrane^2^ and outer membrane vesicles^3^ of *E. coli*. The OmpA proteins in *S. marcescens*, however, are not functionally equivalent to the *E. coli* OmpA protein^4^. The metalloprotease serralysin (~52-55 kDa) is enriched in the *S. marcescens* OMVs, when OMVs are collected from *S. marcescens* cultured at 37℃^5^.

**Methods**

Samples were lysed by RIPA (radioimmunoprecipitation assay) buffer (50 mM Tris (pH 8.0), 150 mM NaCl, 1% NP-40, 0.5% sodium deoxycholate, 0.1% sodium dodecyl sulfate (SDS)) for 15 min at ice, and then boiled for 5 min at 95℃ after adding 5X Laemmli loading dye (250 mM Tris, pH 6.8, 5% β-mercaptoethanol, 0.02% Bromophenol blue, 30% Glycerol, 10% SDS) and cooled at ice to proceed with 13% SDS-PAGE gel separation.

**Reference**

1. Remmert, M., Biegert, A., Linke, D., Lupas, A. N. & Söding, J. Evolution of Outer Membrane β-Barrels from an Ancestral ββ Hairpin. *Mol. Biol. Evol.* **27**, 1348–1358 (2010).
2. Koebnik, R., Locher, K. P. & Gelder, P. V. Structure and function of bacterial outer membrane proteins: barrels in a nutshell. *Mol. Microbiol.* **37**, 239–253 (2000).
3. Thoma, J. *et al.* Protein-enriched outer membrane vesicles as a native platform for outer membrane protein studies. *Commun. Biol.* **1**, 1-9 (2018).
4. Braun, G. & Cole, S. T. DNA sequence analysis of the Serratia marcescens ompA gene: implications for the organisation of an enterobacterial outer membrane protein. *Mol. Gen. Genet.* **195**, 321–328 (1984).
5. Mcmahon, K. J., Castelli, M. E., Vescovi, E. G. & Feldman, M. F. Biogenesis of Outer Membrane Vesicles in Serratia marcescens Is Thermoregulated and Can Be Induced by Activation of the Rcs Phosphorelay System. *J. Bacteriol.* **194**, 3241–3249 (2012).
